# The pathway to residency in Germany: a survey study to identify factors that impact an international medical graduate from Syria

**DOI:** 10.1186/s12909-022-03582-6

**Published:** 2022-07-01

**Authors:** Rakan Saadoun, Eva-Maria Risse, Leen Sadoun, Yusuf Surucu, Ranim Bittar, Mhd Anas Heshma, Theresa Obermueller

**Affiliations:** 1 Department of Plastic Surgery, University of Pittsburgh, Pittsburgh, PA USA; 2 Ruprecht Karls University Heidelberg, Heidelberg, Germany; 3 University of Damascus, Damascus, Syria; 4 Department of Hand, Plastic and Reconstructive Surgery, Plastic and Hand Surgery, BG Trauma Center Ludwigshafen, University of Heidelberg, Burn Center, Ludwigshafen, Germany; 5 Department of Otorhinolaryngology, Head and Neck Surgery, Charité-Universitätsmedizin Berlin, Corporate Member of Freie Universität Berlin, Humboldt University Berlin, Berlin Institute of Health, Campus Benjamin Franklin, Berlin, Germany

**Keywords:** Germany, Residency, International medical graduate, Foreign medical graduate

## Abstract

**Background:**

The German health care system has recently become an attractive destination for international medical graduates, particularly from developing countries such as Syria. However, there are no studies about the factors that influence the successful entry into the German healthcare system at trainee level.

**Method:**

An anonymous cross-sectional survey was distributed electronically to Syrian medical graduates who successfully entered residency training in Germany. Collected data included demographics and factors that influence entering the residency, such as proven German proficiency and clinical experience in the home country. Hypothesis testing was used to assess the difference between the variables.

**Results:**

A total of 109 participants responded to the survey. Twenty-three (21.1%) subjects completed a medical residency in Syria before moving to Germany, and 46 (42.2%) had no previous clinical experience before moving to Germany. The proven German proficiency of the participants upon arrival in Germany was less than B1 in 39 (35.8%), B1 in 37 (33.9%), and B2 in 33 (30.3%) cases. None of the participants had a language level beyond B2, and 18 (16.5%) had no German knowledge.

The median of months spent in Germany till residency for those with B1 or B2 certificates before moving to Germany (10.5 (6.25–16) months and 8 (5–11) months, respectively) differed significantly from those with German-language skills belowB1 ((21 (14–29) months, *p* < .001). Residency in the home country was not associated with a difference in the median of the months in Germany till entering residency, *p* = 0.84.

**Conclusion:**

A crucial factor influencing the successful entry to the German medical system at the trainee level is the ability to speak German, measured in levels based on the Common European Framework of Reference for languages. A high language skill level is a crucial factor associated with a decrease in time in Germany till entering residency for an international medical applicant. In contrast, previous work experience is not influencing the entry into the German labor market.

**Supplementary Information:**

The online version contains supplementary material available at 10.1186/s12909-022-03582-6.

## Introduction

Germany has suffered from a shortage of physicians over the last decade. Driving factors are an aging workforce, the transition of physicians into other industries, especially the pharmaceutical sector and the emigration of German physicians to Scandinavia and Switzerland for better working conditions [1–5].

Because of this shortage, more unfilled residency positions have become available in Germany for international medical graduates (IMGs) [1, 5].

The IMGs applicant, who wishes to start a residency in Germany, usually starts learning German, which is the official language for education and communication in German hospitals, as their first step to residency in Germany. The language level will be assessed based on the Common European Framework of Reference. This framework includes different language levels (A1, A2, B1, B2, C1, C2). A1 is the beginner level, while C2 stands for being proficient in German like a native speaker. However, certificates according to this framework are available only for the levels B1, B2, C1, and C2. The German language skills below the B1 level cannot be officially certified. Certificates for B1 and B2 levels are the most common ones applicants obtain outside Germany [6].

The German residency system differs from those in the United States (US) or the United Kingdom (UK). In comparison, entering the residency in these latter two countries includes an application through a central system (National Resident Matching Program in the US and National Health Service in the UK) [2, 7, 8]. Future residents apply for residency directly to the hospital in Germany rather than through a central service. If applicant’s credentials match the hospital's expectations, they will be invited to an interview. Usually, the applicant would get to know the decision within a few weeks after the interview. The beginning of the residency training is not limited to a specific time of the year and the applicants can start their residency training anytime they agree with the hospital. However, the lack of a central process for application makes it challenging to issue a descriptive statistical report yearly that involves the main characteristics of all the applicants who obtained a residency spot in contrast to the US and UK where these reports are available to the public [2, 7, 8].

So, applicants will have difficulty answering questions like, would a residency in my home country benefit me in assuring residency in Germany? Should I learn German in my home country? And to how many hospitals should I send applications to?

The answers to all these questions have a crucial impact on the individual applicant, who usually comes from developing countries such as Syria with way less average income and living costs than usual in Europe.

Germany recently attracted many Syrian doctors. With more than 5000 physicians, this population is the second biggest group of physicians after Germans working in Germany [5, 9].

We tried to explore the characteristics of the applicants of this group in terms of the time they spent in Germany before entering the residency, the language level they assured back home before moving to Germany, and the number of applications they sent before obtaining their first residency spot. We hypothesized that German proficiency and finishing a residency training in Syria are not associated with successfully entering the residency in Germany within one year of arrival.

## Methods

### Participants and data collection

A cross-sectional survey was distributed anonymously to define factors associated with successfully obtaining a residency spot. The study cohort includes Syrian physicians currently working in Germany. Physicians who have never worked as a resident in Germany or who went to medical school inside the European Union or Switzerland were excluded. The participants were informed that the survey was voluntary and anonymous.

The collected data included age, gender, the place of the medical school attended, clinical experience including homeland residency training, proven German proficiency before moving to Germany, time from arriving in Germany to entering a residency training, the ability to assure income through non-medicine related jobs, the ability to obtain support from the German government bodies, attending an observership, the estimated cost of the entire journey to enter the residency and the specialty at which the participant joined his residency training.

### The survey

The questionnaire was designed on feedback from Syrian medical graduates to address the most critical questions for this group when assessing the feasibility of performing their medical specialist training in Germany. A pilot study was performed on 15 Syrian medical graduates who practice medicine in Germany. The survey was found to be of suitable length and clarity, so no additional changes were enacted. Chronically based, we divided the survey into two sections. The first one focuses on the preparation period in Syria (clinical experience and German language courses) before moving to Germany. The second section inquires information about the activities in Germany (German courses, observerships, costs, visa status, first specialty spot, and the duration) before entering the first specialty training. The survey was distributed on two different random days in March and July 2021 through the Facebook group for Syrian doctors in Germany, representing the biggest virtual platform for all Syrian medical graduates in Germany. Participants were asked at the beginning of the survey to confirm that they are currently practicing medicine in Germany. A translation of the survey in English is provided in the supplements.

### Statistical analysis

The respondents were stratified by their proven German language levels, as evident by a certificate before moving to Germany and whether they completed residency training in Syria. Categorical variables are presented as counts and percentages, while the continuous variables are presented as a mean (standard deviation) or median (interquartile range). Differences between the various groups were tested using the Chi-square and Fischer exact tests for categorical variables. In contrast, the continuous variables were tested with one-way ANOVA and the Kruskal–Wallis tests. Multiple comparisons were performed using independent samples of Kruskal–Wallis’s test. A *p-value* of less than 0.05 was considered statistically significant. We performed a univariate binary logistic regression to determine the odds of entering residency within 12 months after arriving in Germany. The one-year timeframe was chosen as the dependent variable because this is the granted duration of a temporary residence permit in Germany to find a trainee spot for jobseeker visa holders according to Sect. 20 of the Act on the Residence, Economic Activity, and Integration of Foreigners in the Federal Territory of Germany [10]. The 10,000 € cut-off point was determined based on the job seeker visa application requirement defined by the German embassy in Beirut [11].

## Results

Initially, 112 physicians completed the questionnaire. Three participants were excluded for meeting at least one of the exclusion criteria, and 109 participants were included in the study. Data for the time in Germany before starting the residency training, the estimated number of applications, and the joined specialty were missed in four, two, and two cases, respectively. The mean age of the participants was 32.7 (standard deviation (SD) = 4.32) years, and 14 (12.8%) of them were female. The vast majority (106 (97.2%)) of the participants completed medical school in Syria, while the rest (3 (2.8%)) studied medicine in Egypt. Twenty-three (21.1%) subjects completed a medical residency in Syria before moving to Germany.

The proven German levels for the participants upon arriving in Germany were less than B1 in 39 (35.8%) cases, B1 in 37 (33.9%), and B2 in 33 (30.3%). None of the participants had a language level beyond B2, and out of those with less than B1, 18 (16.5%) had no German knowledge.

Upon arrival, more women (64,3%) than men (31,5%) had German skills that were tested below B1 level.

However, the median time in months to enter residency in Germany for men 7 (IQR 7–19) was not significantly higher than the median for women 12 (IQR 7–19) at *p* = 0.985*.*

Most of the participants, 90 (82.6%), attended an observership in a medical facility after arriving in Germany, and the hospital paid them in 13 (14.4%) of the cases. In 53 (58.9%) cases, the length of the observership was one to four months. Forty (36.7%) participants assured financial aid from German bodies to support starting their residency in Germany, such as funding German courses through the Federal Office for Migration and Refugees. Out of those who received financial support, 17 (42.5%) submitted a request for asylum in Germany.

Thirteen (11.9%) participants obtained a mini job outside the healthcare sector to ensure their living costs in Germany. Eight (61.5%) of these thirteen participants submitted an asylum request.

Regardless of the German language level or other factors, the number and proportions of subjects who entered residency were 56 (53.3%), 31 (29.6%), and 14 (13.3%) within one, two, and three years after arrival in Germany, respectively.

The estimated number of submitted applications for residency differed widely among the participants and was in the median 50 (IQR 10–100). Similarly, the estimated cost for the whole project, from decision making to entering the residency in Germany, was also very variable; however, 57 (52.3%) of participants entered their first residency spot with less than 10,000€ (Tables 1 and 2).

**Table 1:** Characteristics of the participants stratified by proven German level

**Variable**	**Total** N	**Proven German Level before moving to Germany**			***P-value***
		**Less than B1** N (%)	**B1** N (%)	**B2** N (%)	
	109	39 (35.8)	37 (33.9)	33 (30.3)	
mean age in years (SD)	32.7 (4.3)	33.2 (4.9)	32.4 (4.4)	32.4 (3.4)	0.45
Median time in Germany till starting residency in months (IQR)	12 (7–20)	21 (14–29)	10.5 (6.25–16)	8 (5–11)	< .001
Median estimated number of applications (IQR)	50 (10–100)	30 (5–100)	100 (25.5–175)	25 (2–67.5)	< .019
Gender					
Male	95 (87.2)	30 (76.9)	36 (97.3)	29 (87.9)	0.025
Female	14 (12.8)	9 (23.1)	1 (2.7)	4 (12.1)	
Completed medical school in Syria					
Yes	106 (97.2)	39 (100)	37 (100)	30 (90.9)	NA
No	3 (2.8)	0 (0)	0 (0)	3 (9.1)	
Completed residency in Syria					
Yes	23 (21.1)	10 (25.6)	6 (16.2)	7 (21.2)	0.6
No	86 (78.9)	29 (74.4)	31 (83.8)	26 (78.8)	
Attended observership in Germany					
Yes	90 (82.6)	31 (79.5)	32 (86.5)	27 (81.8)	0.71
No	19 (17.4)	8 (20.5)	5 (13.5)	6 (18.2)	
Estimated costs in Germany					
≥ 10,000€	52 (47.7)	23 (59)	15 (40.5)	14 (42.4)	0.21
< 10,000€	57 (52.3)	16 (41)	22 (59.5)	19 (57.6)

**Table 2:** Characteristics of the participants stratified based on completing a home country residency training

Variable	Total	Completed residency in Syria		*P-value*
		**No**	**Yes**	
	109	86	23	
Mean age in years (SD)	32.7 (4.32)	31.30 (3.28)	37.48 (3.28)	< .001
Median time in Germany till starting residency in months (IQR)	12 (7–20)	12 (7–19)	11 (9–22)	0.84
Median estimated number of applications, count (IQR)	50 (10–100)	30 (9.25–137.50)	50 (25–90)	0.93
Gender				
Male	95 (87.2)	73 (84.9)	22 (95.7)	0.29
Female	14 (12.8)	13 (15.1)	1 (4.3)	
Completed medical school in Syria				
Yes	106 (97.2)	83 (96.5)	23 (100)	1
No	3 (2.8)	3 (3.5)	0 (0)	
Attended observership in Germany				
Yes	90 (82.6)	69 (80.2)	21 (91.3)	0.35
No	19 (17.4)	17 (19.8)	2 (8.7)	
Estimated cost				
> 10,000€	52 (47.7)	43 (50)	9 (39.1)	0.35
≤ 10,000€	57 (52.3)	43(50)	14 (60.9)	

In a logistic regression model that accounts for completing residency training in Syria, an applicant with a B1 certificate from their home country was found to be at increased odds of entering the residency within one year after arriving in Germany (OR 6.938, 95%CI: 2.4–20.58, *p* < 0.001), when compared to an applicant with less than B1 level in German. Moreover, in this model, a candidate with a B2 certificate has almost a 20-folds increase in odds of entering residency within one year compared to the same reference group (OR 21.046, 95%CI: 6.261–70.754, *p* < 0.001) (Table 3).

**Table 3:** Multivariate Logistic Regression Analysis for odds of entering residency within one year after arriving in Germany

Variable	Odds ratio (95%CI)	*p*-value
Completed residency in Syria	1.341 (0.438–4.108)	0.607
B1 certificate from home country	6.938 (2.4–20.058)	< 0.001
B2 certificate from home country	21.046 (6.261–70.754)	< 0.001

**Fig. 1:**
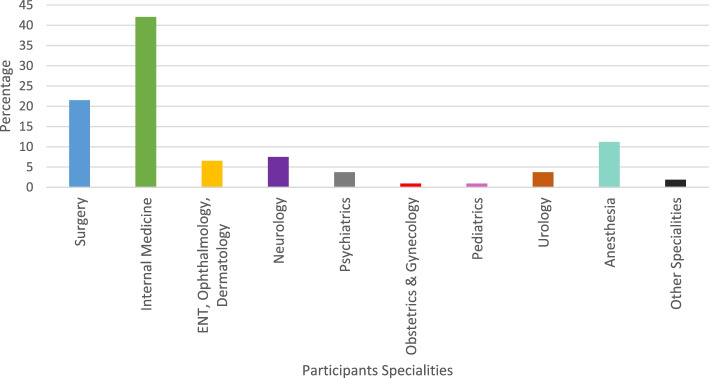
Distribution of the entered residency programs by the participants

We found that seven (6.4%) participants have assured a residency in Otolaryngology and Ophthalmology, and four (3.7%) subjects in Urology **(**Fig. 1).

## Discussion

**Fig. 2:**
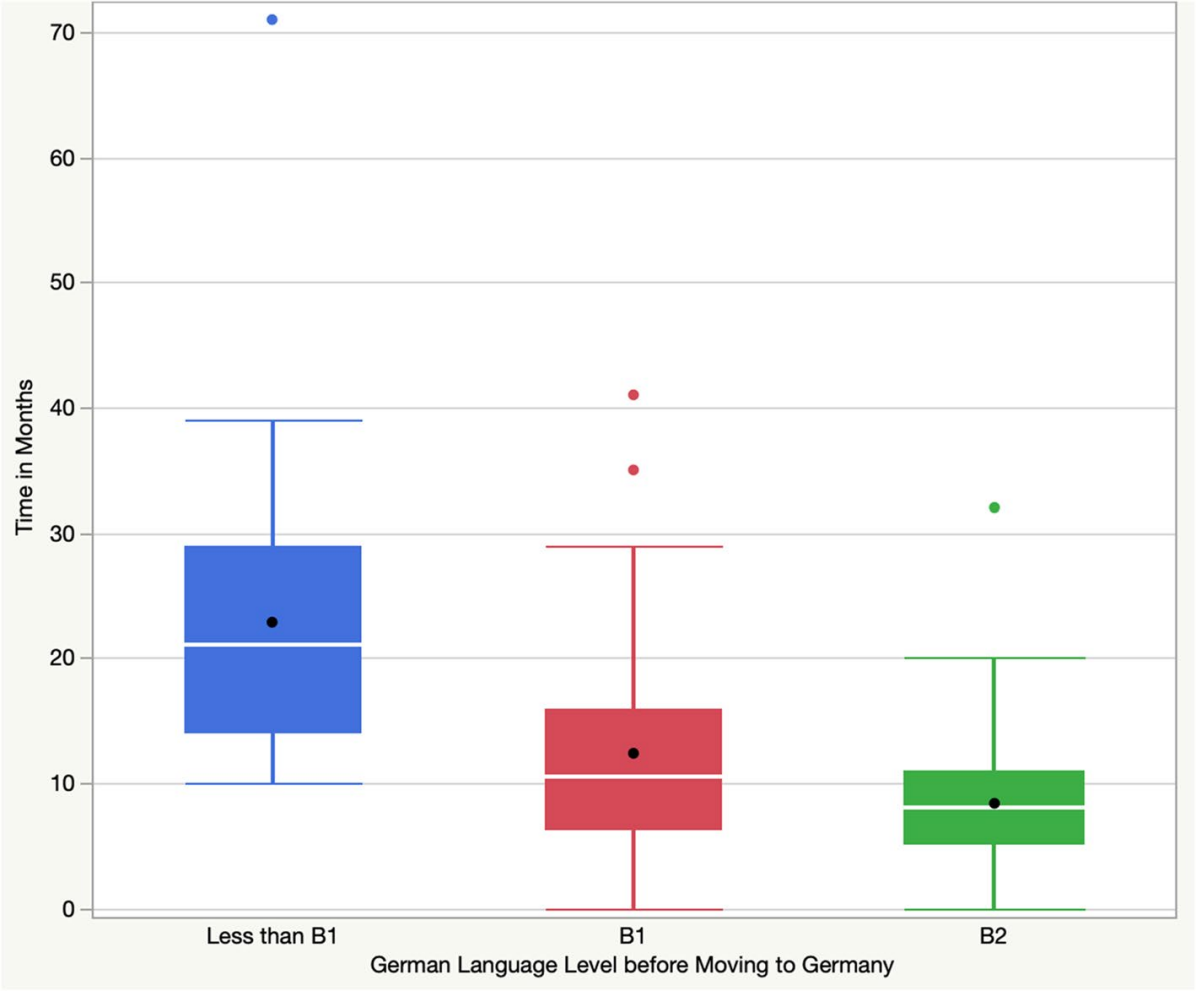
Box and whisker plot of time (in months) spend in Germany from arrival to entering residency for the groups that were stratified based on the proven German language level before moving to Germany. The boxes represent the interquartile range (IQR) with whiskers extending to 1.5 times the IQR. The median is marked with a solid white line. Outliers are marked with a solid circle and the mean with solid black points. Note that roughly 75% of participants with a B2 and level and 50% with a B1 certificate could assure residency within 12 months. In contrast, only less than 25% of participants with a German level of less than B1 could enter residency within one year after arrival in Germany

This survey study is the first to explore factors that impact the entering of IMGs into residency training in Germany. We found that the proven German language level before leaving the home country expressed by B1 or B2 certificates is the most decisive factor for a quick entry into residency in Germany (Fig. 2).

Previous Norwegian studies stressed the importance of the language proficiency of an IMG for interacting with colleagues and patients [12, 13]. Our study shows that candidates who obtained B1 or B2 certificates before moving to Germany were significantly quicker in obtaining residency spots. Moreover, even after accounting for completing a residency in Syria, applicants with proven B1 and B2 levels have an almost 6-folds and 20-folds increase in odds of entering their clinical training within one year, respectively. No previous studies from Germany or neighboring countries addressed the association between proven language level in the home country and the time for entering residency. However, a data linkage study that analyzed the performance of the working IMGs in the UK found the score of the International English Language Testing System (IELTS) to correlate positively with the performance of this group in an examination that requires a minimum of one year of clinical training [14]. Another similar work from the UK found the high IELTS score to predict less practice of fitness events [15]. The findings of those two studies from the UK suggest a more successful pathway with an increased level of English proficiency [14, 15].

In our work, the apparent difference in odds between the groups based on their German proficiency level could be attributed to the fact that a resident’s job in the first year in Germany is mainly taking a medical history, performing a physical exam, and obtaining informed consent rather than doing surgeries or procedures. These tasks require powerful verbal skills to ensure a patient-physician relationship [2, 12, 13].

Consequently, hospitals may prefer a candidate with more vital lingual skills over one with more surgical and medical experience. So, it may be recommended that IMG candidates who wish to assure a training spot in Germany as soon as possible concentrate on improving their language skills and obtaining the B1 or B2 certificates.

Our study also showed that completing residency training in Syria was not significantly associated with quicker entering residency programs or having less difficulty finding the training spot, as expressed by the estimated number of applications sent by the applicant. There are no previous studies from Germany to compare with. However, Chandra et al. reported similarly, in a retrospective study about IMG who successfully entered US residency in neurosurgery, no association between matching to a highly ranked residency program and prior residency training in the home country [16].

An IMG who has the final goal to practice in Germany should weigh well the benefit of taking residency in his home country for his final purposes.

Syrian graduates were able to obtain positions in all specialties, including seven (6.4%) participants assured a residency in specialties such as Otolaryngology and Ophthalmology. Similarly, four (3.7%) subjects entered training in Urology**.** Entering these specialties in countries such as the US or Canada as an IMG usually includes a couple of years of research and a very competitive matching process [7, 17–19]. This could make Germany a desirable choice for IMG seeking residency training in these specialties.

Women constitute almost 56% of students in Syrian medical schools [20]. However, the shift in the gender of the participants was clearly towards male physicians, with only 14 (12.8%) female physicians taking part in the survey. There is no apparent reason for the male predominance; however, a rational explanation could be the conservative nature of the Syrian culture that generally doesn’t support the relocation of single women.

Even though our study investigated the IMGs from Syria, the findings of this study may apply to a proportion of IMGs who obtained a medical education outside the European Union that is comparable to the Syrian. However, our results may probably not apply to IMGs from more advanced education systems like the UK, USA, and Canada.

## Limitations

As our study is the first study that addresses these questions for this specific population, there is, unfortunately, no previous literature from Germany to which we can compare our results. The questionnaire was not a validated tool due to the lack of prior literature on this specific group. Additionally, the candidates with a smoother pathway may be more willing to take the survey than the ones who had difficulties assuring a residency spot. However, we addressed the most critical questions for IMG applicants in Germany regarding securing their residency spots as fast as possible to reduce cost and time of uncertainty. The response rate could not be assessed as the participation was voluntary and anonymously over a public link. As the survey was voluntary, it may be prone to sampling bias as accomplished people may be more likely to take a survey that explores their achievement. Future studies on IMGs applying to German residency programs are still needed to identify the most crucial factors that impact the successful application.

## Conclusion

This is a cross-sectional study on Syrian medical graduates who are working in Germany. We found that the certificated German language knowledge in the home country was associated with less time in Germany to enter residency training. Finishing residency in the home country was not associated with a faster track to entering a German specialty training.

## Supplementary Information

**Additional file 1.** The objective of this survey is to examine thefollowing aspects. 

## Data Availability

The data that support the findings of this study are available from the corresponding author on a reasonable request. The data are not publicly available due to the institution’s research privacy policy.

## Abbreviations

IMG(s): International Medical Graduate(s)
UK: United Kingdom
US: United States
IELTS: International English Language Testing System
