# Supplementary material for: The pathway to residency in Germany: a survey study to identify factors that impact an international medical graduate from Syria
Source: BMC Med Educ. 2022 Jul 1;22:513. doi: 10.1186/s12909-022-03582-6 (PMC9248083; doi:10.1186/s12909-022-03582-6)
Supplement: Supplementary file 1 — Additional file 1. The objective of this survey is to examine thefollowing aspects. [file 12909_2022_3582_MOESM1_ESM.docx]

The objective of this survey is to examine the following aspects:

What factors, e.g., language skills or clinical experience, play a role in being successfully offered a position as a resident in Germany?

On average, how long does it take to get a job offer in Germany? Can factors such as language skills and clinical experience lengthen or shorten the time-to-offer in Germany?

What are the overall costs of moving to Germany and settling and starting as a resident?

What is the likelihood of getting financial support from German (medical) organizations or state funding?

Participation is entirely voluntary and anonymous.

1. Can you confirm that you have at least once worked as a resident in Germany?

🞎 Yes

🞎 No

1. Have you studied in Syria?

🞎 Yes

🞎 No

- If not, please name the country where you have finished your medical degree. ___________

1. What is your age? ___________ years
2. Sex

🞎Male

🞎 Female

**Section I: The preparation for the journey in the home country (Syria).**

1. Have you already completed a residency program in Syria?

🞎 Yes

🞎 No

1. How long was your clinical experience in Syria after finishing your medical school?

🞎 none

🞎 less than one year

🞎 one year

🞎 two years

🞎 three years

🞎 four years

🞎 five years

🞎 more than five years

1. Have you already acquired any German language skills in Syria?

🞎 Yes

🞎 No

**Section II: The preparation for residency in Germany**

1. What was your proven language level when you came to Germany?

🞎 A1

🞎 A2

🞎 B1

🞎 B2

🞎 C1

🞎 C2

1. When did you arrive in Germany?

___________ date (DD/MM/YYYY)

1. When did you take up your first residency position in Germany?

___________ date (DD/MM/YYYY)

1. Did you have the opportunity to work in a paid job in Germany until you got your first position as a resident?

🞎 Yes

🞎 No

1. Have you done an observership for at least one month before starting your residency?

🞎 Yes

🞎 No

- If yes, was it a paid observership ?

🞎 Yes

🞎 No

- If yes, how long was this observership in months?

🞎 one month

🞎 two months

🞎 three months

🞎 four months

🞎 five months

🞎 six months

🞎 seven months

🞎 eight months

🞎 nine months

🞎 ten months

🞎 eleven months

🞎 twelve months

🞎 more than one year

🞎 more than two years

1. Please give an assessment of how much money you invested to move to Germany until the time you started as a resident.

🞎 100-3000 euro

🞎 3001-6000 euro

🞎 6001-10000 euro

🞎 10001-15000 euro

🞎 15001-20000 euro

🞎 20001-25000 euro

🞎 more than 30000 euro

1. Do you receive any financial support from a German organization (Federal Employment Agency, Jobcentre... etc.) along the way to your first position as a resident? Examples could be the financing of language courses, preparation for German medical examinations, maintenance costs, etc.)

🞎 Yes

🞎 No

1. Have you applied for asylum in Germany?

🞎 Yes

🞎 No

1. In which specialty was your first position as a resident in Germany?

🞎 Trauma surgery and orthopedics

🞎 Internal medicine

🞎 Surgery

🞎 Ophthalmology

🞎 Dermatology

🞎 Otolaryngology

🞎 Radiology

🞎 Neurology

🞎 Gynecology and obstetrics

🞎 Pediatrics

🞎 Psychiatry

🞎 Pathology

🞎 Microbiology

🞎 Radiotherapy

🞎 General Medicine

🞎 Occupational Medicine

🞎 Urology

🞎 Anesthesia

🞎 Another specialty

15. Please give an assessment, how many applications did you send

to get your first job offer.

___________ (count)
